# Supplementary material for: Identification of photoperiod-induced specific miRNAs in the adrenal glands of Sunite sheep (Ovis aries)
Source: Front Vet Sci. 2022 Jul 22;9:888207. doi: 10.3389/fvets.2022.888207 (PMC9354845; doi:10.3389/fvets.2022.888207)
Supplement: Supplementary file 1 [file Data_Sheet_1.ZIP › Supplementary file/miRDeep2 file.zip!/miRDeep2 file/Know_Structure/SP42A2/oar-mir-374a.pdf]

miRBase precursor : oar-mir-374a
Total read count : 27102
oar-miR-374a read count : 22989
remaining reads : 4113

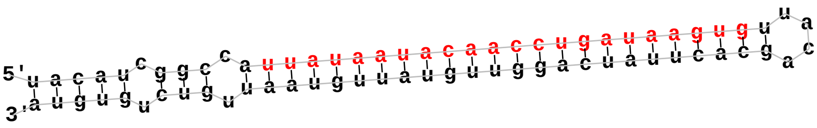

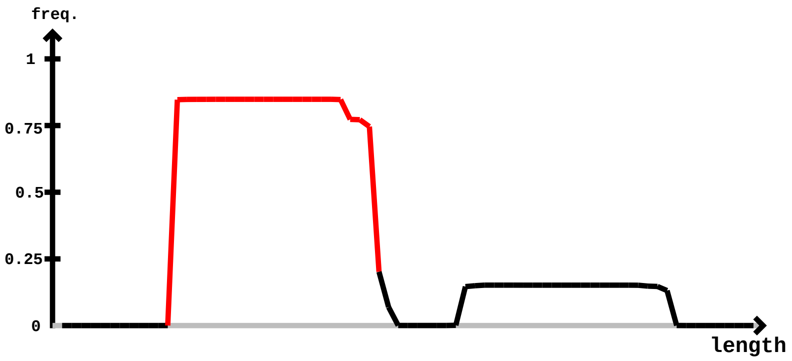


```
                  oar-miR-374a
5'- uacaucggccauuauaauacaaccugauaagugguuacagcacuuaucagguuguauuguaauugucugugua   -3'    exp
    (((((.(((.((((((((((((((((((((((((....)))))))))))))))))))))))).))).)))))   reads      mm         sample
    ..........auuauaauacaaccugauaagu........................................   1          0          seq
    ..........auuauaauacaaccugauaagug.......................................   1          0          seq
    ...........Auauaauacaaccugau............................................   1          1          seq
    ...........uuaCaauacaaccugau............................................   1          1          seq
    ...........uuauaauacaaccugau............................................   26         0          seq
    ...........uuaAaauacaaccugaua...........................................   18         1          seq
    ...........uuauaauacaaccugauU...........................................   32         1          seq
    ...........uCauaauacaaccugaua...........................................   2          1          seq
    ...........uuauaauacaaccugaua...........................................   1898       0          seq
    ...........uuauaauacaaccugGua...........................................   8          1          seq
    ...........uuauaauacaUccugaua...........................................   2          1          seq
    ...........uuauUauacaaccugaua...........................................   6          1          seq
    ...........uGauaauacaaccugaua...........................................   2          1          seq
    ...........uuaCaauacaaccugaua...........................................   29         1          seq
    ...........Cuauaauacaaccugaua...........................................   17         1          seq
    ...........uuauaauacaaccugauaa..........................................   32         0          seq
    ...........uuauaauacaaccugauaag.........................................   687        0          seq
    ...........uuaCaauacaaccugauaag.........................................   9          1          seq
    ...........uuauaauacaaccugGuaag.........................................   4          1          seq
    ...........uuaCaauacaaccugauaagu........................................   179        1          seq
    ...........uGauaauacaaccugauaagu........................................   1          1          seq
    ...........uuauaauacaaccugauaagu........................................   14558      0          seq
    ...........uuauaauacaaccugauaagug.......................................   3548       0          seq
    ...........uuaCaauacaaccugauaagug.......................................   43         1          seq
    ...........uuauaauacaaccugauaagugu......................................   1849       0          seq
    ...........uuauaauacaaccugauaaguguu.....................................   2          0          seq
    ............uauaauacaaccugauaag.........................................   1          0          seq
    ............uauaauacaaccugauaagu........................................   13         0          seq
    ............uauaauacaaccugauaagug.......................................   1          0          seq
    ............uauaauacaaccugauaagugu......................................   11         0          seq
    .............auaauacaaccugauaagu........................................   2          0          seq
    .............auaauacaaccugauaagugu......................................   4          0          seq
    ..............uaauacaaccugauaaguguu.....................................   1          0          seq
    ..............uaauacaaccugauaaguguuacagcacu.............................   1          0          seq
    ..............uaauacaaccugauaaguguuacagcacuuauc.........................   1          0          seq
```

oar-miR-374a

uacaucggccauuauaauacaaccugauaaguguuacagcacuuaucagguuguauuguaauugucugugua			
...............aauacaaccugauaaguguuacagcac..............................	1	0	seq
...............aauacaaccugauaaguguuacagcacu.............................	2	0	seq
...............aauacaaccugauaaguguuacagcacuu............................	1	0	seq
....acuuaucagguuguauugua............	4	0	seq
....acuuaucagguuguauuguaau..........	8	0	seq
....acuuaucagguuguauuguaauu.........	9	0	seq
....cuuaucagguuguauugu.............	8	0	seq
....cuuCucagguuguauugua............	3	1	seq
....cuuaucagguuguauugua............	83	0	seq
....cuuaucagguuguauuguaa...........	36	0	seq
....cuuaucagguuguauuguaau..........	397	0	seq
....cuuaucagguuguauuAuaau..........	1	1	seq
....cuuaucagguuguauuguaauu.........	3398	0	seq
....cuuaucagguuAuauuguaauu.........	3	1	seq
....cuuaucagguuguauuguaauug........	4	0	seq
....uuaucagguuguauugua............	1	0	seq
....uuaucagguuguauuguaau..........	8	0	seq
....uuaucagguuguauuguaauu.........	81	0	seq
....uuaucagguuAuauuguaauu.........	1	1	seq
....uaucagguuguauuguaauu.........	59	0	seq
....uaucagguuguauuguaauug........	2	0	seq
....uaucagguuguauuguaauugu.......	1	0	seq
